# Preclinical development of a Pfs230-Pfs48/45 chimeric malaria transmission-blocking vaccine

**DOI:** 10.1038/s41541-021-00383-8

**Published:** 2021-10-12

**Authors:** Susheel K. Singh, Jordan Plieskatt, Bishwanath K. Chourasia, Vandana Singh, Karin Lövgren Bengtsson, Jenny M. Reimer, Renate C. van Daalen, Karina Teelen, Marga van de Vegte-Bolmer, Geert-Jan van Gemert, Matthijs M. Jore, Michael Theisen

**Affiliations:** 1 Department for Congenital Disorders, Statens Serum Institut, Copenhagen, Denmark; 2 Centre for Medical Parasitology at Department of Immunology and Microbiology, University of Copenhagen, Copenhagen, Denmark; 3 PATH’s Malaria Vaccine Initiative, Washington, WA USA; 4 Novavax AB, Uppsala, Sweden; 5 Department of Medical Microbiology, Radboud University Medical Center, Nijmegen, Netherlands

**Keywords:** Biotechnology, Immunology, Microbiology, Medical research

## Abstract

The *Plasmodium falciparum* Pfs230 and Pfs48/45 proteins are leading candidates for a malaria transmission-blocking vaccine (TBV). Previously, we showed that a Pfs230–Pfs48/45 fusion protein elicits higher levels of functional antibodies than the individual antigens, but low yields hampered progression to clinical evaluation. Here we identified a modified construct (ProC6C) with a circumsporozoite protein (CSP) repeat-linker sequence that enhances expression. A scalable and reproducible process in the *Lactococcus lactis* expression system was developed and ProC6C was successfully transferred for manufacturing under current Good Manufacturing Practices (cGMP). In addition, a panel of analytical assays for release and stability were developed. Intact mass spectrometry analysis and multiangle light scattering showed that the protein contained correct disulfide bonds and was monomeric. Immunogenicity studies in mice showed that the ProC6C adsorbed to Alhydrogel^®^, with or without Matrix-M^TM^, elicited functional antibodies that reduced transmission to mosquitoes and sporozoite invasion of human hepatocytes. Altogether, our data support manufacture and clinical evaluation of ProC6C as a multistage malaria-vaccine candidate.

## Introduction

Malaria is a vector-borne disease caused by *Plasmodium* parasites, of which *Plasmodium falciparum* causes the highest morbidity and mortality worldwide. *P. falciparum* parasites are transmitted from one person to another by mosquitoes. Malaria transmission-blocking interventions have therefore largely focused on vector control, but the increase in insecticide resistance calls for new tools. Malaria transmission-blocking vaccines (TBV) hold the potential to block malaria transmission in the population, thereby contributing to malaria elimination. Such vaccines aim to produce specific antibodies against functionally important proteins expressed during parasite development in the mosquito.

Three proteins, Pfs25, Pfs230, and Pfs48/45, are currently lead candidates for a TBV (reviewed in^1,2^). Of these, Pfs230 and Pfs48/45 are expressed during gametocyte development in humans as well as in mosquitoes where they form a protein complex on the surface of the *P. falciparum* gamete^3^. Humans can develop naturally acquired immunity against gametocytes through natural exposure and the presence of antibodies against Pfs48/45 and Pfs230 has been associated with transmission-reducing activity (TRA)^4^. While others have focused on Pfs230 Domain 1^5^, here we focused on the N-terminal Pro-domain, absent of cysteines to prevent scrambled disulfide bonds, while maintaining some transmission-reducing activity^6,7^. Recently, we showed that affinity-purified human antibodies against Pfs48/45 and Pfs230 exhibited strong TRA in the standard membrane-feeding assay (SMFA)^8^, the gold standard for assessing TRA ex vivo^9–12^. To explore whether there is an additive or synergistic activity between Pfs230 and Pfs48/45 antigens, we have screened a series of chimeric vaccine antigens composed of different Pfs48/45 and Pfs230 domains in preclinical models^13^. In these studies, dual-antigen vaccines elicited higher levels of functional antibodies than the corresponding single-antigen vaccines. Importantly, antibody-depletion experiments demonstrated that specific antibodies against the individual Pfs230 and Pfs48/45 domains each contributed to functional activity in the SMFA strongly supporting the use of chimeric proteins in vaccine development.

From a manufacturing perspective, however, protein-expression yields of the aforementioned Pfs230–Pfs48/45 chimeras were low and not optimized^13^. Assuming that complexity of disulfide-bond formation might reduce the overall secreted-protein yield of the chimera, we decided to focus on the N-terminal Pro domain of Pfs230 as it does not contain cysteine residues, expresses at a reasonable yield of 30 mg/L alone^13^, and because it elicits complement-dependent TB activity in the SMFA^6^. We further hypothesized that appropriate spacer sequences between the constituent antigens of this chimera might increase overall folding and solubility, resulting in an increased yield. Naturally occurring multidomain proteins often contain one or more linker peptides that act as spacers between functionally distinct domains^14^. Such linkers may also contain intrinsically disordered protein sequences that are thought to form highly dynamic extended structures allowing the connecting domains to freely twist and rotate^15^.

In the present study, we tested a panel of linker sequences to increase yields of a chimeric Pro-6C construct. This panel included natural linker sequences derived from the *P. falciparum* circumsporozoite protein (*Pf*CSP) because of this protein’s distinct structural motifs^16,17^, some of which elicit protective antibodies^18–20^. Furthermore, we describe expression and purification of the selected construct from bench scale (1 and 5L) to support evaluation in phase I clinical studies. Finally, to complement the strategy for the generation of drug substance presented here, we also propose the planned Drug Product configuration, i.e., adsorbed to Alhydrogel^®^ and with the inclusion of the saponin-based adjuvant Matrix-M^TM^.

## Results

### Expression of multidomain TBV antigens in *L. lactis*

**Fig. 1:**
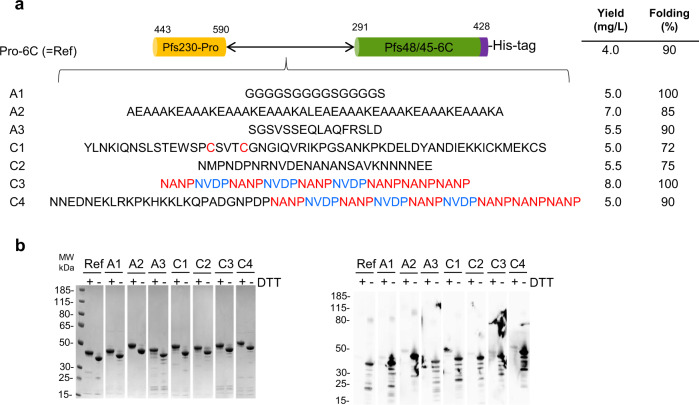
Design and expression screening of Pro-6C chimeras. **a** Schematic representation of Pro-6C and different linker sequences. A panel of linker sequences (A1–C4) were inserted between the two domains. **b** Coomassie blue-stained 4–12% polyacrylamide gel of purified proteins from 0.5 L-scale fermentation. Ref: Pro-6C. **c** Immune-blotting analysis of the same gel using the conformational reduction-sensitive mAb45.1 as primary antibody. All blots and gel are derived from the same experiment and processed in parallel. Composite images are presented in panels (**a**) and (**b**) from original images files provided in Supplementary Figure S4 and S5, respectively.

To increase overall proper folding and yield, we have optimized antigen design by inserting a set of spacer sequences between the Pfs230-Pro and Pfs48/45-6C domains (Fig. 1a). The linkers (of different length, composition, and secondary structure) are based on either published spacer sequences^14^ or defined structural domains of *Pf*CSP: The N-terminal region, the central repeat region with the NANP and NVDP protein motifs, and the C-terminal region containing two disulfide bonds (Supplementary Fig. S1). Each construct was transformed into *L. lactis* MG1363 and small test expressions were performed. For comparison, expression of the Pro-6C fusion protein lacking a linker sequence^13^ was assessed in parallel. All recombinant clones produced a secreted recombinant protein and reacted with the transmission-blocking (TB) mAb45.1 that binds to the conformational epitope I in the Pfs48/45-6C domain (Supplementary Fig. S2). Proper folding of the Pfs230 Pro-domain could not be assessed as no functional mAbs against this domain have been described.

### Production of TBV chimera in a bioreactor

To obtain a more accurate estimate of expression yields, all constructs (his-tagged) were expressed in 0.5-L bioreactors. Each construct was purified from the culture supernatants using His-Trap columns. Fractions containing high quantities of target protein, as determined by SDS-PAGE analysis, were pooled and applied to an ion-exchange chromatography column to separate monomeric and covalently linked multimeric protein species. The monomeric protein fraction of each construct was analyzed by SDS-PAGE and immune blotting with conformational mAb45.1 (Fig. 1b). The overall yield and folding of each chimera relative to immune-purified reference material is indicated (Fig. 1a). Insertion of a linker sequence increased the overall yield of the chimera compared with Pro-6C up to approximately 2-fold in the case of linkers A2 and C3.

### Immunogenicity of TBV chimeras in mice

**Fig. 2:**
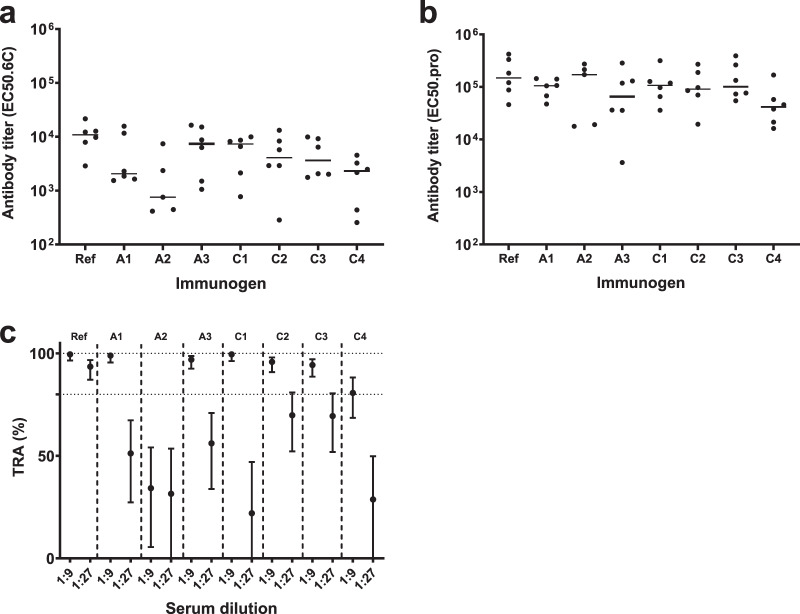
Immunogenicity and functional activity of Pro-6C chimeras. Groups of mice (*n* = 6) were immunized with 10 µg of Pro-6C constructs with alum. Day-56 serum was tested for antibody reactivity on ELISA plates coated with Pfs48/45-6C (**a**) and Pfs230-Pro (**b**). Antibody titers (dots represent individual mice) are expressed as EC50 values. Horizontal lines represent median values. **c** Functional activity of pooled sera (1:9 and 1:27 dilution) in the standard membrane-feeding assay (SMFA). Reported values and 95% confidence intervals (bars) are determined by general linearized mixed models and used oocyst-count data from two independent SMFA experiments with 20 mosquitoes per condition and experiment. Transmission-reducing activity is calculated by comparing with a nonserum control included in each SMFA.

It is well established that the folding of recombinant Pfs48/45 strongly influences its ability to elicit functional antibodies^21,22^. The immunogenicity of all chimeras was therefore investigated by immunizing outbred CD-1 mice three times at three-week intervals. Two weeks after the last injection, mice were bled and specific antibodies were assessed by ELISA on plates coated with the constituent antigens, Pfs48/45-6C or Pfs230-Pro. All constructs elicited domain-specific antibodies comparable to those obtained with Pro-6C, except for Pro-A2-6C (lower levels of 6C-specific antibodies) and Pro-C4-6C (lower levels of Pro- and 6C-specific antibodies) (Fig. 2a,b). Antisera were then tested for functional activity in the SMFA. In agreement with antibody titers, antisera against Pro-C4-6C and Pro-A2-6C showed lower TRA, while antisera raised against the other constructs showed similar levels of TRA (Fig. 2c). Since Pro-C3-6C showed high yield and induced a strong functional antibody response, we selected this construct for further preclinical development and renamed it ProC6C.

### Process development for ProC6C

While the constructs were previously His-tagged to facilitate early evaluation, we replaced the His tag in ProC6C with a C-tag, which consists of four amino acids (EPEA), to be more amenable for cGMP manufacturing^23^. The strain expressing ProC6C was grown at 30 °C as described in detail^24^.

**Fig. 3:**
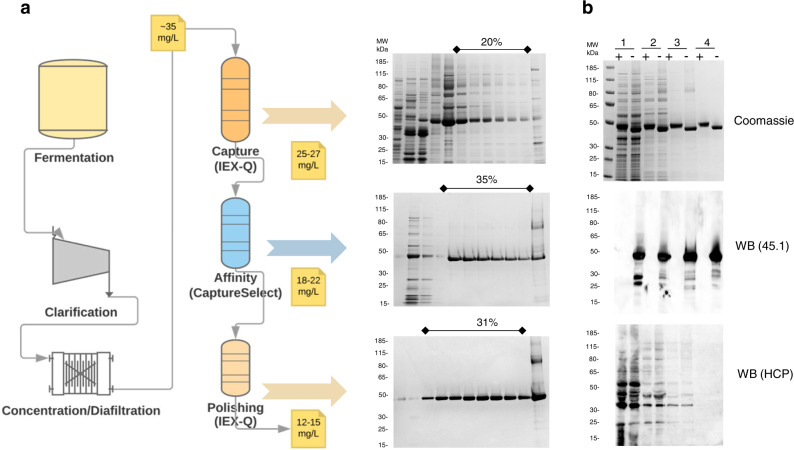
Production of ProC6C in *L. lactis*. **a** Schematic representation of fermentation and three purification steps of ProC6C protein with associated yields and recovery of purified protein. Coomassie blue-stained 4–12.5% polyacrylamide gel inset with elution fractions (concentration B buffer); *upper panel* HiPrep Q HP column, *middle panel* Capture selectXL column, and *lower panel* HiPrep Q HP. The sample was loaded without a reducing agent. **b** Analysis of purified ProC6C by SDS-PAGE; *upper panel*; 1 diafiltrate, 2 HiPrep Q HP column, 3 Capture selectXL column, 4 HiPrep Q HP column purified ProC6C. An immune-blot analysis of the same gel shown in the *upper panel* using mAb45.1 and anti-*L. lactis* antibodies (detection of HCP) in the *middle* and *lower panels*, respectively. Protein was loaded in each lane with (+) or without (−) DTT (10 mM). The sizes (kDa) of the molecular mass markers are indicated. All blots and gel are derived from the same experiment and processed in parallel.

ProC6C was purified from cell culture media by (1) a capturing step using anionic exchange chromatography, (2) an affinity-chromatography step using a CaptureSelect™ column, which has a high affinity toward the C-tag, and (3) a polishing step using anionic exchange chromatography to obtain a pure preparation of monomeric ProC6C (Fig. 3a). Column parameters for the purification of ProC6C are listed in Supplementary Table S1. With this three-step purification procedure, the overall yield from cell culture medium to the final product was approximately 12 mg/L (Table 1), equal to an overall 40% recovery rate from culture supernatant. The purity and folding of each step were determined by SDS-PAGE, and western blot with mAb45.1 and a rabbit antiserum against *L. lactis* host-cell proteins (HCP) (Fig. 3b). Most of the loss during purification was observed in the initial capture step.

**Table 1:** Mass Balance summary of ProC6C.

Step	1 L Total protein (mg/L)	1 L ProC6C (mg/L)	5 L Total protein (mg/L)	5 L ProC6C (mg/L)
Harvest supernatant	22300^a^	30–35	22800^a^	30–40
Diafiltration/buffer exchange	680	30–35	645	38
Capture (Q)	390	25–27^b^	360	26–30^b^
HCP removal (Capture select)	40	18–22^b^	35	18–22^b^
Polishing (Q)	15	12–15^c^	12	10–12^c^

^a^Total protein is over-estimated due to the presence of Cysteine and Cystine in the medium.

^b^Amount of ProC6C in the eluate.

^c^Amount of ProC6C monomer in the eluate.

### Physical and biochemical characterization of ProC6C

To evaluate the quality of purified ProC6C, we determined whether it was structurally intact. The molecular mass of nonreduced ProC6C was 36308.54 ± 2 Da as determined by LC–MS, which corresponds well to the predicted value of 36315.33 Da. Reduction of ProC6C resulted in an increase in mass of 6 Daltons to 36314.59 ± 2 Da, which corresponds to three disulfide bonds that are present in the Pfs48/45-6C domain of the chimera (Table 2). Collectively, these results indicate that the secreted soluble protein was intact, contains three paired cysteines, and does not contain post-translational modifications.

**Table 2:** Characterization of ProC6C.

Test	Method	1L consistency 1	1L consistency 2	1L consistency 3	5 L lab-scale
pH	pH meter	8.0	8.0	8.0	8.0
Color and appearance	Visual observation	Colorless	Colorless	Colorless	Colorless
Endotoxin	LAL	11 EU/mg	9.0 EU/mg	12.5 EU/mg	17 EU/mg
Residual host-cell protein by ELISA	Immunoenzymatic Assay	<1%	<1%	<1%	<1%
Protein content	A_280_/Nanodrop	0.65 mg/mL	0.58 mg/mL	0.60 mg/mL	0.8 mg/mL
Accurate quantification of the total protein	AAA analysis	0.68 mg/ml	ND	ND	0.81 mg/ml
Identity and integrity (SDS-PAGE)	SDS-PAGE both nonreduced and reduced	Major monomer band	Major monomer band	Major monomer band	Major monomer band
Identity (WB)	Both nonreduced and reduced Western Blot against monoclonal antibodies 45.1	Major monomer band	Major monomer band	Major monomer band	Major monomer band
Purity RP-HPLC	RP-HPLC	98.3%	>97.8%	>96.9%	>97.3%
Identity, integrity and purity (SE-HPLC)	SE-HPLC	97.3% monomer	98.5% monomer	98.0% monomer	96.5% monomer
Intact mass spectrometry analysis of full-length protein	Outsourced: Intact mass spec (LC–MS/MS) under nonreducing and reducing conditions	Nonreduced (36308.54 Da) Reduced (36314.599 Da)	ND	ND	Nonreduced (36308.54 Da) Reduced (36314.599 Da)

**Fig. 4:**
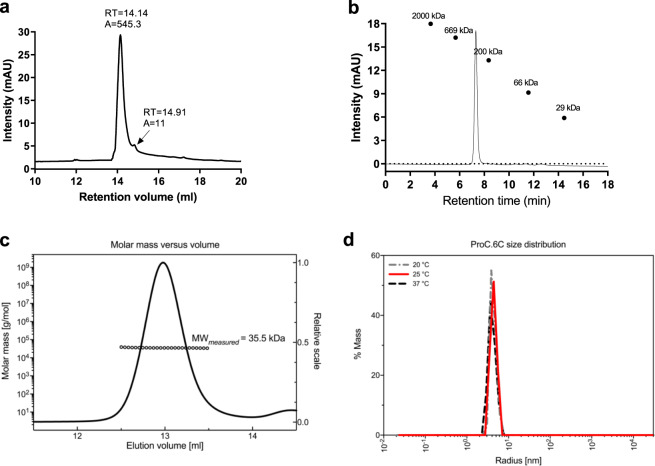
Characterization of ProC6C. **a** Reversed-phase (RP) HPLC. Reversed-phase HPLC–UV chromatogram recorded following analysis of purified ProC6C. The peak at 14.14 ml corresponds to monomeric ProC6C antigen. **b** Size-exclusion chromatography (SEC) HPLC analysis. SEC-HPLC analysis was performed under native conditions in a phosphate buffer pH 6.7 to determine the amount of monomer in the sample. **c** SEC-MALS analysis. ProC6C was injected onto a Superdex 200 Increase 10/300GL column and the change in refractive index as a function of protein concentration was used to compute the molar masses. The solid line plotted on the *right axis* corresponds to the change in refractive index from the SEC column. The right axis is normalized to the greatest magnitude across the chromatogram data, i.e., to the monomer peak of ProC6C. The molar masses across the eluting peak are plotted as open circles on the *left axis* (molar mass). The average molecular mass is indicated. **d** Distributions of hydrodynamic radii obtained from dynamic light scattering (DLS) on ProC6C at three different temperatures.

Reversed-phase HPLC was performed to assess the purity of the final ProC6C protein. ProC6C eluted as a single main peak (Fig. 4a). The presence of few minor peaks indicates a homogeneous population of ProC6C protein species with a relative purity of 98.3% (Fig. 4a). Analytical size-exclusion chromatography (SEC) analysis revealed that ProC6C eluted as a single peak (Fig. 4b). Using a standard of a range of globular proteins, the calculated molecular weight was approximately 300 kDa via SEC, indicating the molecule is largely intrinsically unstructured with a large overall hydrodynamic radius. Multi-angle light scattering coupled to size-exclusion chromatography (SEC-MALS) was used to determine the absolute molecular weight of soluble ProC6C. ProC6C eluted as a main monodisperse peak at 12.9 ml (Fig. 4c) with an average molecular mass of 35.5 kDa, indicating that ProC6C is monomeric in solution and present at the proper molecular weight. The aberrant migration pattern of ProC6C by SEC might be explained by the relative abundance of intrinsically disordered sequences in the Pro-domain, similar to earlier observations for another 6C fusion construct^13^. Furthermore, to analyze the behavior of ProC6C proteins in solution and in particular to probe for aggregation and stability, dynamic light scattering (DLS) was performed. ProC6C consists of a homogeneous population of mainly monomeric protein species with a low percentage of polydispersity (<23%) and an average radius of 5.1 nm in both pre- and post-thermal elevation, as demonstrated by DLS (Fig. 4d).

### Process consistency and protein stability

To demonstrate the consistency of the production process, the whole process, including the upstream and downstream steps, was repeated three times at 1L scale. The characterization of purified ProC6C from the three consistency runs is summarized in Table 2 and demonstrated that the developed process was reproducible. Planned cGMP-release testing and tentative specifications are shown in Table 3. The final purified protein from process-consistency runs served as reference-standard material during cGMP and technology transfer. The ProC6C is monitored for long-term stability under frozen conditions (<−50 °C) and, with analysis still ongoing, has not shown any change on SDS-PAGE, in mAb45.1 recognition or in monomeric state after one year (Supplementary Fig. S3).

**Table 3:** ProC6C cGMP proposed release assays.

Attribute	Test	Analytical test method	Proposed specifications
Biochemical	Appearance	Visual assessment USP<1790>	Clear, colorless solution free of particles
Biochemical	pH	Potentiometric pH USP<791>	7,5–8,5
Biochemical	Total Protein Concentration	SE-HPLC	≥0.4 mg/ml
Biochemical	Endotoxin	LAL	≤100 EU/mg
Identity	Protein ID	Western blot	Positive identification and predominant band at expected molecular weight
Identity/purity	ProC6C full-length MW	SDS-PAGE	Predominant band at expected molecular weight ≥85%
Identity/purity	ProC6C full-length	SE-HPLC	≥85% monomer
Potency	Biological Activity	ELISA Folding	≥85%
Residual/purity	Residual DNA	Quant-it	10 pg/µL to 100 ng/µL
Residual/purity	Host cell protein	*L. lactis* WB	<1% w/w
Residual/purity	Boron/borate	ICP-AES	TBD
Safety	Pyrogen	Rabbit pyrogen testing USP<151>	USP<151>
Safety/purity	Microbial enumeration	Bioburden USP<61>	USP<61>

### Immunogenicity of ProC6C formulations

**Fig. 5:**
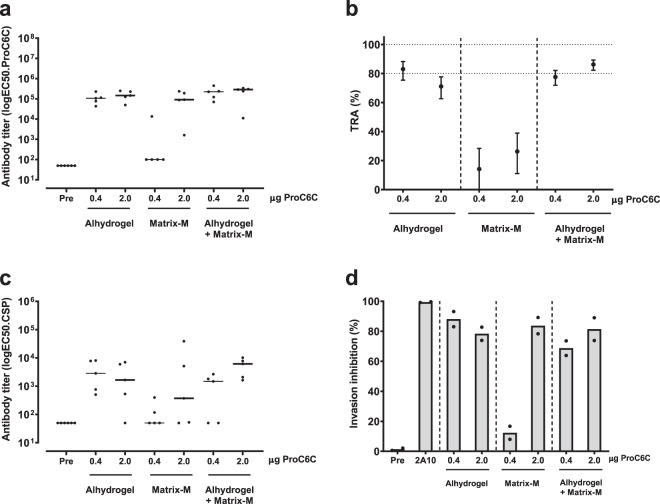
Immunogenicity and functional activity of ProC6C vaccine formulations. Groups of mice were immunized with ProC6C formulated with Alhydrogel^®^ alone, Matrix-M adjuvant alone, and Alhydrogel® together with Matrix-M adjuvant. **a** Levels of vaccine-specific antibodies in individual mice are shown as midpoint titers. Midpoint titers below 50 are reported as 50. Bars represent median values. **b** Pooled sera were tested in SMFA at 1:9 dilution. Reported values and 95% confidence intervals (bars) are determined by general linearized mixed models and used oocyst count data from two independent SMFA experiments with 20 mosquitoes per condition and experiment. Transmission-reducing activity is calculated by comparing with a nonserum control included in each SMFA. **c** Levels of CSP-specific antibodies in individual mice are shown as midpoint titers. Midpoint titers below 50 are reported as 50. Bars represent median values. **d** Inhibition of sporozoite invasion of human hepatocytes (HC-04) by 1:20-diluted heat-inactivated mice sera. Dots are means of three independent replicates in each experiment, and bars are means of the two independent experiments. Preimmune mouse serum (Pre) and 100 µg/mL anti-CSP antibody 2A10^46^ were included as negative and positive controls, respectively.

To identify a potent ProC6C vaccine formulation, we tested ProC6C in formulations with Alhydrogel® and Matrix-M^TM^ adjuvant. Matrix-M is a saponin-based adjuvant, purified from the *Quillaja saponaria molina* tree, with a good safety record in humans^25^ and demonstrated to enhance the immunogenicity of our other Pfs48/45-based TBV candidate, R0.6C, in mice^26^. A suboptimal vaccination regimen was used to detect differences in immunogenic properties between vaccine formulations as well as any potential dose-sparing effect of the Matrix-M adjuvant. Groups of outbred CD-1 mice were immunized twice at four-week interval with 0.4-µg or 2.0-µg doses of ProC6C formulated with Matrix-M adjuvant, Alhydrogel or both combined. Two weeks after the last immunization, mice were bled and vaccine-specific antibodies were quantified by ELISA (Fig. 5a). When ProC6C was formulated in Matrix-M adjuvant alone, negligible levels of specific antibodies were achieved in the 0.4 μg group. On the other hand, no clear dose-dependent antibody response was observed for the other two formulations, suggesting that these formulations have a dose-sparing effect (Fig. 5a). The functionality of the induced antibodies was then assessed in SMFA (Fig. 5b). Only very low transmission-reducing activity was observed for the Matrix-M adjuvant-alone group. Importantly, pooled sera from groups that received 0.4 µg or 2.0 µg of ProC6C adjuvanted with Alhydrogel and Alhydrogel with Matrix-Mreduced transmission ~80%, demonstrating that both formulations are viable candidates for clinical evaluation. These results also align with our clinical development program and previously reported study for the Pfs48/45 malaria TBV, where the Matrix-M adjuvant increased immunogenicity and allowed dose-sparing of R0.6C with Alhydrogel^26^.

To investigate whether the CSP repeat linker of the ProC6C construct elicited functional antibodies, we first quantified antibody responses in CSP ELISA^27^. Formulations with Alhydrogel and Alhydrogel with Matrix-M induced high levels of antibodies, while responses in mice immunized with Matrix-M alone showed lower responses (Fig. 5c), in line with antibody responses against the whole construct (Fig. 5a). We next tested whether the antibodies were functional in an invasion-inhibition assay with *P. falciparum* sporozoites and human hepatocytes. We observed low levels of inhibition by antibodies that were induced with 0.4 µg of ProC6C formulated with Matrix-M (Fig. 5d), in agreement with the low levels of CSP antibodies in this group (Fig. 5c). Interestingly, the other formulations were able to induce antibodies that strongly inhibited sporozoite invasion of human hepatocytes (Fig. 5d). Altogether, these data demonstrate that ProC6C formulated with Alhydrogel with or without Matrix-M-elicited antibodies that block parasite transmission to mosquitoes and prevent sporozoite invasion of human hepatocytes.

## Discussion

Recently, *L. lactis* has gained importance in the manufacturing of recombinant proteins for subunit vaccine development^28–30^. *L. lactis* has proved to be particularly useful for the production of disulfide-bonded proteins^31^. Here *L. lactis* proved indeed highly suitable for the production of cGMP-compliant ProC6C expressed as a soluble secreted protein with apparent proper cysteine connectivity. Importantly, the final product elicited high levels of functional antibodies in preclinical models justifying further clinical studies.

To enable clinical development of a Pfs230–Pfs48/45 chimera, we have redesigned a vaccine antigen and subsequently optimized the production process to increase overall yield and folding. This chimera replaced the carrier protein (the glutamate-rich protein “GLURP”) of our other TBV candidate (R0.6C^24,26^) with the Pro-domain of Pf230. We selected the Pro-domain of Pfs230 with the C-terminal cysteine-rich domain of Pfs48/45 because (i) these protein domains are established targets of functional antibodies^6,8,13,32^ and (ii) the Pro-domain does not contain cysteine residues that could interfere with disulfide formation within the 6C domain^13^. Since linker sequences are known to affect folding and solubility of fusion proteins^14^, we have here screened a panel of linkers with different properties such as flexibility, length, and secondary structure. We identified a CSP-based peptide (C3) as optimal linker for separation of the Pfs230-Pro and Pfs48/45-6C domains, which includes six NANP- and three NVDP repeats. Our finding that the *Pf*CSP repeats can serve as a spacer in the Pfs230–Pfs48/45 fusion protein may be related to the finding that the NANP repeat region of CSP adopts an extended conformation, creating an elongated and flexible molecule^17^. Whether increasing the length of C3 by incorporating additional repeats might further increase yield is not known. However, considering the immune dominance of the CSP repeats^18^, additional copies might negatively affect antibody responses against the respective Pfs230- and Pfs48/45 domains.

Once the optimal construct (termed ProC6C) had been defined, the production process was optimized at bench scale to prepare for cGMP. The robustness of the bench-scale process was confirmed in three consistency runs with identical upstream and downstream processes. The final product was found to be consistent, pure, stable, and immunogenic. Intact mass analysis of ProC6C confirmed the expected molecular weight and the presence of three disulfide bonds in the final product. SEC-MALS analysis demonstrated that the final batch of ProC6C forms a homogeneous population of monomeric protein. The final bench-scale process described here is identical to how the process will be performed in the GMP suite.

Building on past experiences with R0.6C^26^, we have investigated the immunogenicity of ProC6C in formulations containing Alhydrogel and/or Matrix-M adjuvant. While Alhydrogel is one of the most commonly used adjuvants for human use, it may also have its limitations in terms of its ability to enhance and/or shape antigen-specific immune responses^33,34^. It is therefore important to investigate adjuvants such as Matrix-M^35^, which is a promising new technology with an excellent safety profile in humans^25,36–38^. Here we demonstrated that ProC6C adsorbed on Alhydrogel and supplemented with Matrix-M adjuvant elicited high levels of functional antibodies even at the very low dose (0.4 µg), consistent with the results obtained with R0.6C^26^. In contrast, ProC6C in Matrix-M adjuvant alone did not elicit equivalent high levels of functional antibodies. The exact reason for this observation is unknown, but we note that the Matrix-M adjuvant is highly efficient for the generation of protective antibodies against the malaria vaccine candidate R21^39^. Based on the data presented in this ProC6C study and in the R0.6C preclinical study^26^, the vaccine antigen may be poorly immunogenic alone (i.e., when not formulated with Alhydrogel). Adsorption to Alhydrogel may result in multimeric forms of the antigen and enhance presentation to immune cells that are activated and recruited to the injection site and draining lymph nodes by Matrix-M. When the antigen is not adsorbed to Alhydrogel, it may not be taken up by immune cells, resulting in reduced immune responses. It is well documented that Alhydrogel enhances Th2-type responses; thus, a combination of both adjuvants may be optimal for the production of antibody responses against ProC6C. The study of the biochemical interactions between antigen, Matrix-M, and Alhydrogel are planned in parallel to the clinical development to better understand the impact on immune responses. Although it is not yet fully understood how Matrix-M adjuvant achieves its stimulatory effects, prior studies have suggested that the Matrix-M adjuvant is likely to improve not only the magnitude of immune response but also the duration and durability of the response—tangible points central to a successful malaria TBV^40^ as discussed in detail^26^.

Whether anti-ProC6C antibodies inhibit development of naturally circulating genetically diverse *P. falciparum* strains remains to be investigated. Given the limited antigenic variation observed in the Pfs230-Pro and Pfs48/45-6C domains^2^, we anticipate that ProC6C induces broadly neutralizing antibodies. Indeed, antibodies elicited by a similar Pfs48/45-based vaccine R0.10 C blocked transmission of parasite isolates from geographically distinct locations^20^.

The primary objective of this study was to create an efficacious TBV-vaccine candidate. However, the CSP linker in our ProC6C vaccine candidate also induced high levels of antibodies that prevent infection by inhibiting sporozoite-hepatocyte invasion. Combining CSP and the sexual-stage antigens Pfs230 and Pfs48/45 is attractive since it could reduce the risk of infections and onward transmission to other individuals via mosquitoes simultaneously. Adding anti-infection properties to a TBV may improve community acceptance. Furthermore, combining both activities may result in synergistic effects, as demonstrated by Sherrard-Smith et al. in a rodent malaria model-based population study^41^.

In conclusion, we here report a process for the cGMP production of a multistage vaccine, ProC6C, as well as potent human-use formulations to advance this candidate to first-in-human clinical evaluation.

## Methods

### Preparation of constructs

All the chimeric constructs are based on the *L. lactis* pSS1 plasmid vector^32^. Codon-optimized Pro-6C-His containing different linkers (Fig. 1a and Supplementary Fig. S1) between Pro and 6C was synthesized by (GeneArt^®^ Life Technologies, Germany) and cloned into pSS1 with the help of *BamH*I/SalI cloning sites. For the development of Ni^++^ independent purification of the selected constructs, His tag was replaced with C tag (EPEA) by PCR. All the constructs were verified by sequencing and subsequently transformed into *L. lactis* MG1363 (Bioneer A/S, Denmark) by electroporation as described^42^. Briefly, competent *L. lactis* cells were thawed on ice and 40-µl cells were mixed with 2 µl of DNA in a microcentrifuge tube. The mixture was then transferred to an ice-cold electroporation cuvette and pulsed using the following settings: voltage, 2.0 kV; capacitance, 25 µF; resistance, 200 ohms; and time constant of 4–5. Following discharge, 0.96 mL of ice-cold SGM17 media is added to the cuvette, transferred to a microcentrifuge tube, and incubated at 30 °C for 2 h. The suspension was plated and incubated at 30 °C for up to three days for colony screening.

### Screening, fermentation and protein purification

Screening for expression of chimeric constructs was performed as previously described^24^ with slight modifications. Briefly, *L. lactis* MG1363, containing chimeric Pro-6C constructs with linker, was grown overnight at 30 °C in 5 ml of LAB medium broth (3.5% yeast extract, 0.05 mM FeSO_4_ 7 H_2_O, 2.5 μM CaCl_2_, 2.65 μM MgCl_2_, 0.5 mM citric acid 2H_2_O, 1.38 mM ammonium sulfate, 14.7 mM sodium acetate, and 20 mM KH2PO4 buffer pH 7.4) containing 1 μg/ml erythromycin, 5% glucose, and 4% disodium glycerophosphate. Culture supernatants were clarified by centrifugation at 9000 g for 20 min. Analysis of all the constructs was performed by Coomassie-stained SDS-PAGE gel and Western blotting with mAb45.1. Fermentation of *L. lactis* MG1363/chimeric constructs was performed in a 0.5-L-scale fermenter at 30 °C with gentle stirring (150 rpm). Cell-free culture filtrates were concentrated fivefold and buffer-exchanged into HEPES buffer (20 mM HEPES, 50 mM NaCl, pH 8.0, supplemented with 15 mM imidazole) using a Quix-Stand Benchtop system (Hollow fiber cartridge with cutoff at 10,000 Da, surface area 1400 cm^2^, GE Healthcare, Sweden) followed by filtration through a Nalgene Rapid-Flow Sterile Disposable filter units with PES membrane 0.22-μm pore size. Proteins were purified on a 5-ml His-Trap HP column (GE Healthcare, Sweden) followed by an anionic exchange column as described previously^24^. Purified proteins containing monomers with the highest amount of mAb45.1-reactive protein were concentrated by a VIVA spin column 10-kDa cutoff (GE Healthcare, Sweden), and kept in 20 mM HEPES, 300 mM NaCl, and 1 mM EDTA, pH 8.0, at −80 °C until use.

### SDS-PAGE and Western blot

Samples were diluted with 6X SDS (sodium dodecyl sulfate, Sigma-Aldrich) sample buffer, heated for 10 min at 98 °C, and loaded onto NuPAGE Novex 4–12% Bis-Tris precast gels (Invitrogen, USA) according to the manufacturer’s instructions. Gels were run at 150–200 V for 35–50 min in 1X MOPS SDS running buffer and stained with Coomassie-staining procedures.

Following SDS-PAGE, proteins were transferred to nitrocellulose membrane (Bio-Rad, USA) and blocked in 1% skim milk in TBS containing 0.05% Tween 20 (TBST) at room temperature for 1 h. Primary antibody, conformational, and reduction-sensitive Rat mAb 45.1 was used at 0.5 µg/ml in TBST and incubated for 1 h at room temperature. The membranes were washed with TBST (three times for 5 min) and secondary antibody (1:4000 dilution) of goat anti-rat IgG–HRP conjugated (Cat # 31470, Invitrogen, USA) in TBST was incubated at room temperature for 1 h. The membranes were again washed with TBST (three times for 5 min), developed using HRP kit (SERA CARE, USA).

All blots and gel are derived from the same experiment and processed in parallel. Raw image files for gels and Western blot images are provided in Supplementary materials (Supplementary Figure S4-S7).

### Production of ProC6C at the bench scale

Fermentation of *L. lactis* MG1363/ProC6C construct was performed as previously described^32^ with minor modifications. Briefly, a 15-ml culture tube containing 5 mL of basic lab medium with 4% disodium glycerophosphate was inoculated with 100 µl of working cell bank and incubated for 4–6 h at 30 °C, until culture OD_600_ reached ≥1.0. The fermenter (BIOFLO 310, New Brunswick Scientific) containing 1 L of fermentation medium was inoculated with 0.4 ml of preculture. After 4 h of inoculation, the fermenter was supplied with 50% glucose continuously at the rate of 8 ml/h to maintain 5% glucose in the medium, until the end of the fermentation. The pH was maintained at 6.5 ± 0.2 using 2 M NaOH. Cultivation was carried out at 30 °C with gentle stirring (150 rpm) for 15–18 h, until an OD600 of 12–15 was reached. After 15–18 h of growth, the bulk cell mass was removed by centrifugation (9000 × *g*, 4 °C, 30 min). The culture supernatant was concentrated 5-fold and buffer exchanged in buffer (20 mM HEPES, 5% glucose, 50 mM Sod. borate, 10 mM l-arginine, and 1 mM EDTA, pH 6.5) using a Quix-Stand Benchtop system (hollow-fiber cartridge with cutoff at 10,000 Da, surface area 1400 cm^2^, GE Healthcare, Sweden) followed by filtration through a Nalgene Rapid-Flow Sterile Disposable filter units with PES membrane 0.22-μm pore size.

For the isolation of ProC6C from cell culture media a 3-step purification procedure: (1) capturing by IEC on a HiPrep Q HP column (GE Healthcare, Sweden), (2) removal of HCP on a CaptureSelect™ C-tagXL column (ThermoFisher, USA), and (3) separation of the monomer fraction by IEC on a HiPrep Q HP column was used. ***First step****.* The first step was optimized to capture 80–90% of the target antigen, ProC6C, with minimal binding of unwanted HCP. To ensure maximum recovery from the capturing step, the loading density was maintained at 20 mg/mL of resin or less. The clarified concentrated and buffer-exchanged fermentation supernatant was applied to a HiPrep Q-HP (16/10) column, washed with five column volumes (CVs) of 50 mM NaCl in Buffer A followed by 8 CVs of 100 mM NaCl in the same buffer (Supplementary Table S1). Bound material was eluted with 12 CVs of 200 mM NaCl and fractions containing the desired protein were pooled and diluted 8-fold with Buffer B (Supplementary Table S1) to bring the conductivity below 8 mS/cm for application to the subsequent column. ***Second step****.* The diluted eluate from the capturing step was then applied onto a CaptureSelect™ C-tagXL prepacked 3 × 5 ml column-flow rate 2.5 mL/min. The column was washed with 10 CVs of 140 mM MgCl_2_ in Buffer B and bound protein was eluted with 10 CVs of 700 mM MgCl_2_ in the same buffer (Supplementary Table S1). Fractions containing high concentration of ProC6C were pooled, resulting in a mixture of monomeric and multimeric forms of ProC6C. ***Third step****.* The eluted material from the second column was diluted 8-fold with Buffer C and applied again to a HiPrep Q-HP (16/10). The column was washed with 5 CVs of 150 mM NaCl in Buffer C followed by 8 CVs of 270 mM NaCl in the same buffer, thereby removing residual HCPs and smaller MW protein fragments resulting from proteolytic degradation of the target protein. Bound protein was eluted with 10 CVs of 310 mM NaCl (Supplementary Table S1). Fractions containing a single band of monomeric ProC6C were pooled and diluted in formulation buffer (20 mM HEPES, 5% glucose, 300 mM NaCl, and 1 mM EDTA, pH 8.0) and were stored at −80 °C until further use. Fractions were analyzed by SDS-PAGE and immune blotting with mAb45.1 against Pfs48/45 conformational epitope I. Protein concentration was measured by NanoDrop (A_280_). Column chromatography was performed using an NGC 10 Medium Pressure Chromatography system (Biorad, USA).

### Process scale-up

Fermentation and sample preparation were done as described above. Protein-purification process and column size of each step of purification from 5L scale was performed as mentioned in Supplementary Table S1.

### Protein characterizations and analytical methods

#### Reversed-phase high-performance liquid chromatography (RP-HPLC)

RP-HPLC was performed with minor modifications as described previously^27^. Briefly, RP-HPLC was performed using an Agilent 1100 Series HPLC System (Agilent Technologies, USA) equipped with an Agilent Poroshell 120EC-C18 column, 4 µm 4.6 × 100 mm (Agilent Technologies, USA). About 210 pmol of protein (nonreduced) was injected and eluted (flow rate 1 ml/min) with a linear gradient of 3–95% over 20 min of 0.1% trifluoroacetic acid (TFA), 20% isopropanol, and 70% acetonitrile. The absorbance was measured at 214 nm and chromatographic peaks were integrated by HPLC ChemStation (Agilent Technologies, USA).

### Analytical size-exclusion chromatography (SE-HPLC)

Analytical SE-HPLC was performed as described previously^27^ with slight modification. Briefly, SE-HPLC of purified protein was performed with an Agilent 1100 Series HPLC System (Agilent Technologies, USA) equipped with a TSKgel G3000SWXL (TOSOH Biosciences, USA). An injection volume of ten microliters was used with a mobile phase of 20 mM HEPES, 310 mM NaCl, 5% glucose, and 1 mM EDTA, pH 8.0, at a flow rate of 1 mL/min. The absorbance was measured at 280 nm and chromatographic peaks were integrated by HPLC ChemStation (Agilent Technologies, USA). Protein standards (Sigma Aldrich) were also run using the same conditions mentioned above for sizing of the purified recombinant proteins.

#### Kinetic endotoxin assay

Pierce LAL Chromogenic Endotoxin Quantitation Kit (Thermo Scientific, USA) was used to quantify endotoxin content of purified proteins.

#### Host-cell protein

*L. lactis* HCP kit (Cat # F490, Cygnus Technologies, USA) was used to quantify *L. lactis* HCPs as impurities in the purified proteins as described by the manufacturer.

#### Amino acid analysis (AAA)

AAA was performed in triplicate using 30 μL of sample (30 µg) for each hydrolysis (6 N HCl, 110 °C; 20 h in sealed, evacuated glass tubes). Cysteine and tryptophan were not determined.

#### Mass spectrometry

Accurate molecular mass of ProC6C was measured by LC–ESI–MS under both non-reducing and reducing conditions as previously described^24^. Briefly, 30 pmol of protein were loaded on a C4 precolumn (Acquity UPLC Protein BEH C4 Vanguard, 1.7, 2.1 × 5 mm, Waters, UK) and eluted onto a Q-TOF mass spectrometer (Synapt G2 HDMS, Waters, UK) with a chromatographic gradient. LC–MS data were recorded and analyzed by MassLynx software (Waters, UK). Mass spectra were deconvoluted using the MaxEnt 1 algorithm in the MassLynx software.

#### Analytical size-exclusion high-performance liquid chromatography coupled with multiangle static laser light scattering (SEC-MALS)

A Dionex (Thermo Scientific, USA) HPLC system connected in-line with a UV detector (Thermo Scientific DionexTM Ultimate 3000, MWD3000), a DAWN HELEOS 8+ multiangle laser light-scattering detector, and a Optilab T-rEX (Wyatt Technology Corporation) refractive index detector. Using a Superdex 200 Increase 10/300 GL column (GE Healthcare, Sweden) at room temperature in 20 mM HEPES, 310 mM NaCl, 5% glucose, and 1 mM EDTA, pH 8.0, 100 µl of proteins were injected with a flow rate of 0.5 ml/min. The ASTRA (version 6.1.17) software (Wyatt Technology Corporation, USA) was used to collect UV, refractive index, and light-scattering data. The weight average molecular mass was determined across the elution profile from static light-scattering measurements using ASTRA software and a Zimm model that relates the amount of scattered light to the weight average molecular mass of the solute, the concentration of the sample, and the square of the refractive-index increment (dn/dc) of the sample.

#### Dynamic light scattering (DLS)

DLS experiments were performed on a DynaPro NanoStar cuvette-based instrument (Wyatt Technology Corporation, USA) operated with Dynamics software (version 7.8.1.3). The data were collected at 20, 25, and 37 °C and a total of 10 measurements were performed at each temperature with an acquisition time of 5 s and read interval of 1 s.

### Animals and immunogenicity studies

In the first immunogenicity study, groups of six female CD-1 mice (Janvier Labs, Denmark) were immunized s.c. 3 times at three-week interval with 10 µg of chimeric antigens on 70% Montanide ISA720 (Seppic, France). Mice were sacrificed two weeks after the last immunization and serum was collected for ELISA and SMFA analysis. In the second study, groups of five female CD-1 mice (Charles River, Germany) were immunized i.m. in the right thigh with 50 µl of vaccine (ProC6C), two times with a four-week interval containing 0.4 or 2.0 µg of ProC6C. Alhydrogel (InvivoGen, USA) formulations contained 75 micrograms of Alhydrogel and were mixed by pipetting for 5 min. Matrix-M™ adjuvant (Novavax AB, Uppsala, Sweden) formulations contained 5 micrograms of Matrix-M adjuvant per injection and were mixed by pipetting shortly. Formulations that contained both Alhydrogel and Matrix-M adjuvant were prepared by first adsorbing ProC6C to Alhydrogel as described above and then adding Matrix-M adjuvant. Fourteen days after the last immunization, mice were sacrificed, and serum was collected for ELISA and SMFA analysis.

### Animal study ethics statement

All animal procedures complied with national regulations and ethical regulations for animal testing and research. The experiments included in this study received ethical approval, including the ethics committee of the University of Copenhagen (approval number P 19-308) and the Radboud University Medical Center (approval number 2016-0020).

### Enzyme-linked immunosorbent assay (ELISA)

Antibody responses in mice were quantified by ELISA with plates coated by the vaccine antigen^13^ or full-length CSP^27^. Briefly, microtiter plates (Nunc MaxiSorp, Denmark) were coated with 0.33 µg/ml of recombinant protein in carbonate buffer, pH 9.2, and incubated overnight at 4 °C. Plates were blocked for 1 hr at room temperature (RT) using 3% skimmed milk in 1xPBS with 0.05% Tween 20 (1xPBST), washed twice, and incubated for 1 hr with 100 μl of test serum or a pool of negative control from prebleed mice serum, diluted 1:500 in 1% skimmed milk in 1xPBST. Subsequently, antigen-specific bound antibodies were probed with HRP-conjugated rabbit anti-mouse IgG–HRP (Cat # P0260, DAKO, Denmark) at 1:4000 dilution in 1% skimmed milk in 1xPBST for 1 hr. Plates were washed three times in 1x PBST between each step. ELSA was developed with 100 μl of 3, 3, 5, 5-tetramethylbenzidin (TMB) X-tra substrate (Kem-En-Tec, 4800 A). The color reaction was stopped with 100 μl of 0.2N H_2_SO_4_ and absorbance was measured at 450 nm. Data were collected on a BioSan HiPo MPP-96 microplate reader (BioSan, Latvia). Antibody midpoint titer (EC50) was calculated using sigmoidal curve fitting on GraphPad Prism version 8.4.3 (San Diego, USA).

### Standard membrane feeding assays (SMFA)

Transmission-reducing activity of antibodies was assessed in SMFA. Cultured *Plasmodium falciparum* NF54 gametocytes were fed to *Anopheles stephensi* mosquitoes. Each blood meal contained 150 µL of packed red blood cells with gametocytes, 30 µL of naive human serum containing active complement, and test serum that was diluted in fetal calf serum. After 6–8 days, oocysts in 20 mosquitoes were counted by microscopy. TRA estimates from two independent experiments were generated using generalized linear mixed models (GLMMs) with zero-inflated negative binomial error structure^43,44^. Statistical analyses were performed using R studio (version 3.2.4, The R Foundation, Boston, USA).

### Hepatocyte invasion-inhibition assay

In vitro invasion-inhibition experiments were conducted as previously described^45^. *Plasmodium falciparum* NF54 sporozoites were incubated with heat-inactivated (56 °C, 30 min) mouse sera on ice for 30 min and subsequently incubated with human hepatoma cells (HC-04, MR4-BEI Resources) for 3 h at 37 °C. Supernatant containing noninvading sporozoites was removed and hepatocytes were stained with eBioscience™ Fixable Viability Dye eFluor™ 780 (1:200 dilution, ThermoFisher cat no. 65-0865-14). Hepatocytes were permeabilized with Fixation/Permeabilization buffer (eBioscience) for 30 min. at 4 °C and then washed with Permeabilization buffer (eBioscience). Intracellular sporozoites were stained with 3SP2 antibody that was labeled using Alexa Fluor488 antibody labeling kit (ThermoFisher)^45^, 30 min. at 4 °C. Cells were washed with Permeabilization (eBioscience), fixed with 1% PFA, and analyzed by flow cytometry (Gallios, Beckman Coulter). For each experiment, three independent replicates were analyzed with at least 2000 single live hepatocytes. Invasion inhibition was normalized against a sporozoite only control (0% inhibition).

### Reporting summary

Further information on research design is available in the Nature Research Reporting Summary linked to this article.

## Supplementary information

Supplementary Information

Reporting Summary

## Data Availability

The datasets generated during and/or analyzed during the current study are available from the corresponding author on reasonable request.
