# Supplementary material for: Preclinical development of a Pfs230-Pfs48/45 chimeric malaria transmission-blocking vaccine
Source: NPJ Vaccines. 2021 Oct 12;6:120. doi: 10.1038/s41541-021-00383-8 (PMC8511065; doi:10.1038/s41541-021-00383-8)
Supplement: Supplementary file 1 — Supplementary Information [file 41541_2021_383_MOESM1_ESM.pdf]

Supplementary Material

Preclinical development of a *Plasmodium falciparum* Pfs230-Pfs48/45 chimeric transmission-blocking vaccine

Susheel K. Singh[1,2], Jordan Plieskatt [3], Bishwanath K. Chourasia [1,2], Vandana Singh [1,2], Karin Lövgren Bengtsson[4], Jenny M. Reimer[4], Renate C. van Daalen[5], Karina Teelen[5], Marga van de Vegte-Bolmer[5], Geert-Jan van Gemert[5], Matthijs M. Jore [5,*,#], and Michael Theisen[1,2,*,#]

Supplementary Figures:

MMRKLAILSVSSFLFVEALFQEYQ**C**YGSSSNTRVLNELNYDNAGTNLYNELEMNYYGKQE

NWYSLKKNSRSLGENDDGNNEDNEKLRKPKHKKLKQPADGNPDPNANPNVDPNANPNVDP **C4** **C3**

NANPNVDPNANPNANPNANPNANPNANPNANPNANPNANPNANPNANPNANPNANPNANP

NANPNANPNANPNANP**NVDP**NANPNANPNANPNANPNANPNANPNANPNANPNANPNANP

NANPNANPNANPNANPNANPNANPNANPNANPNKNNQGNGQGHNMPNDPNRNVDENANAN **C2**

SAVKNNNNEEPSDKHIKEYLNKIQNSLSTEWSP**C**SVT**C**GNGIQVRIKPGSANKPKDELDY **C1**

ANDIEKKI**C**KMEK**C**SSVFNVVNSSIGLIMVLSFLFLN

Supplementary Figure S1. Sequence and position of linkers derived from full-length CSP (NCBI reference sequence XM_001351086.1). Shown is the amino acid sequence (in single letter code) of the linker sequences inserted between the Pfs230-Pro and Pfs48/45-6C-domains Pro-6C. The lines below the aa sequence reflect the location of four linkers (C1-C4). The highly conserved KLKQP motif (termed region I), which binds heparin sulfate proteoglycans, is indicated with a line above the sequence. Cysteine residues are indicated in bold. The central repeat region containing the NANP (red) and NVDP (blue) protein motifs are shown.

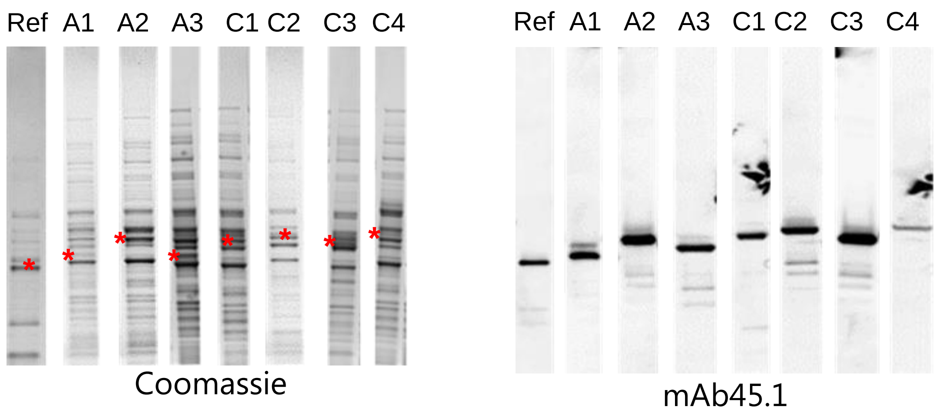

Supplementary Figure S2. Screening for expression of Pro.6C linker constructs: (Left Panel) Coomassie blue-stained 4-12% polyacrylamide gel of supernatants from 5ml culture. Lane1 1: Pro-6C, Lane2: A1, Lane3: A2, Lane4: A3, Lane5: C1, Lane6: C2, Lane7: C3 and Lane8: C4; (Right Panel) Immune blotting analysis of the same gel using the conformational reduction-sensitive mAb45.1 as primary antibody

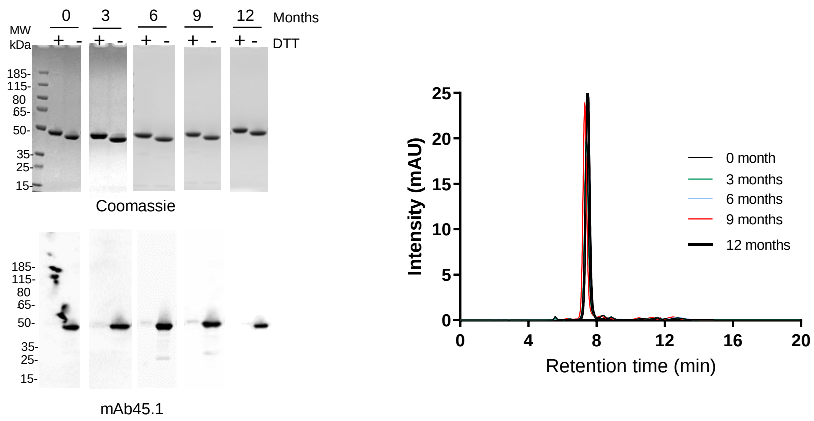

Supplementary Figure S3. Long term stability at -80ºC. (Left Panel) Coomassie blue-stained 4–12.5% polyacrylamide gel; numeric Zero, 3, 6, 9 and 12 correspond to months (*upper panel*); an immune blot analysis of the same gel using mAb45.1 (*lower panel*). (Right Panel) SEC analysis (overlap chromatograms) of samples: Representative SEC chromatograms of ProC6C protein eluted contains the majority of the monomer.

Gel 1:

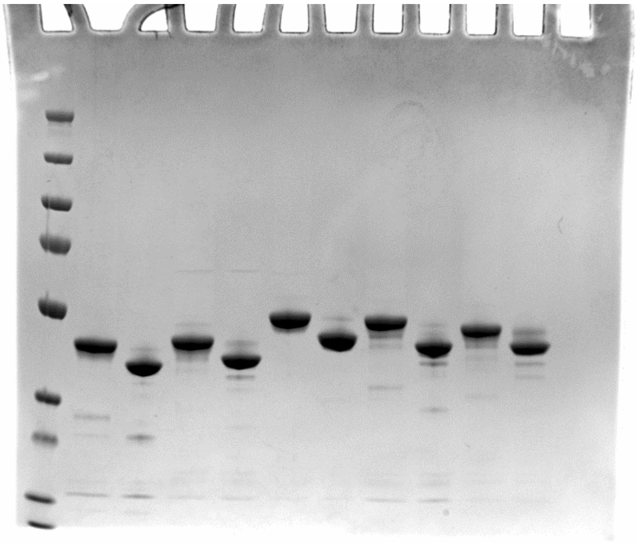

Gel 2:

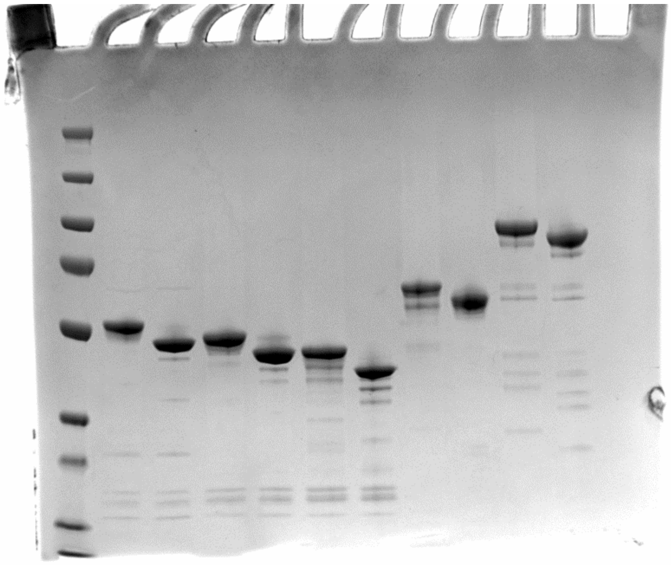

Supplementary Figure S4. Raw original image files for Figure 1b (SDS-PAGE)

Blot 1:

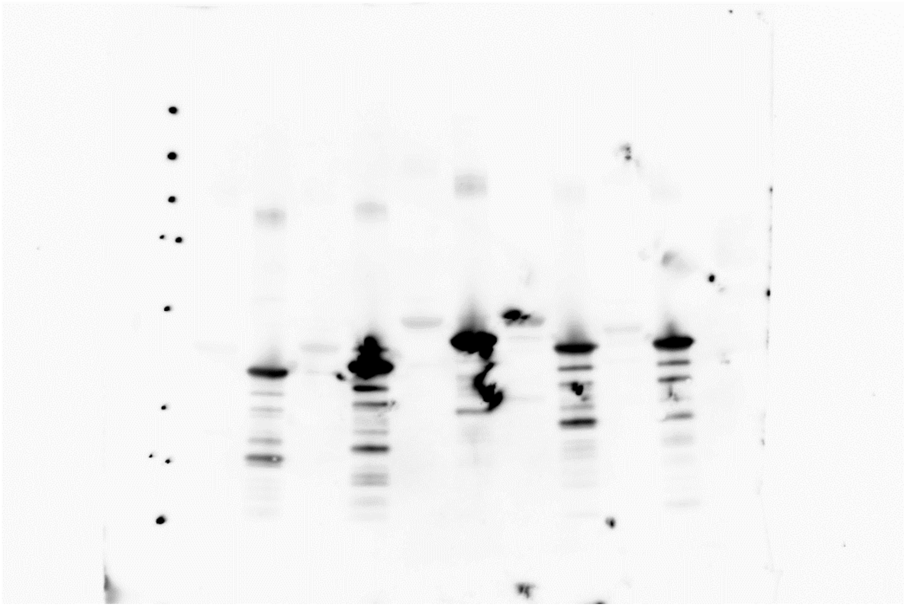

Blot 2:

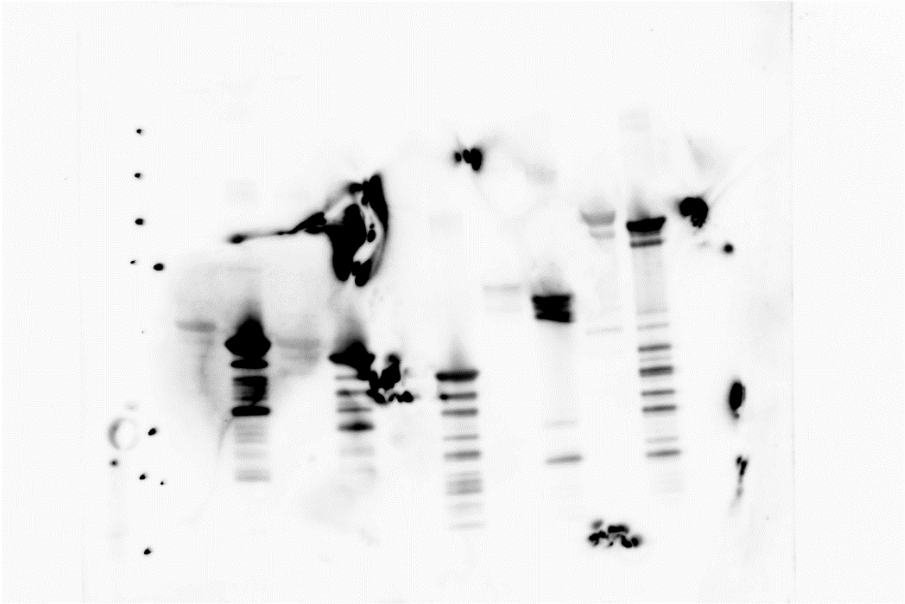

Supplementary Figure S5. Raw original image files for Figure 1b (Western blots)

Gel 1:

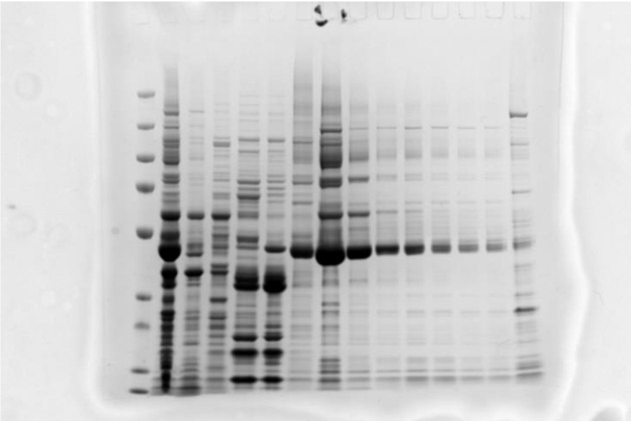

Gel 2:

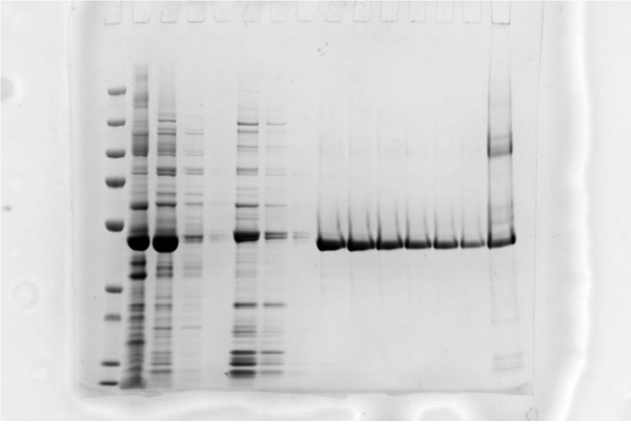

Gel 3:

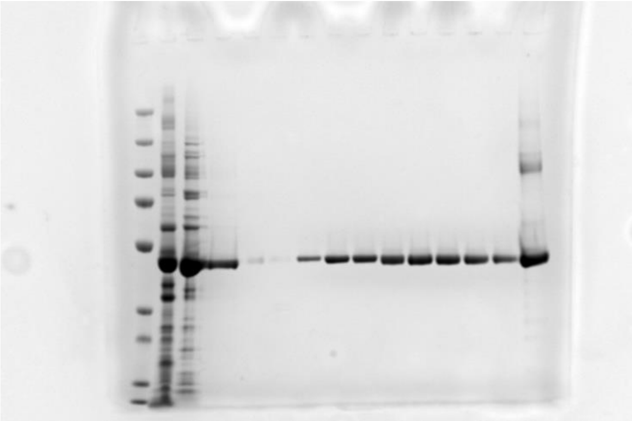

Supplementary Figure S6. Raw original image files for Figure 3a (SDS-PAGE)

Gel 1:

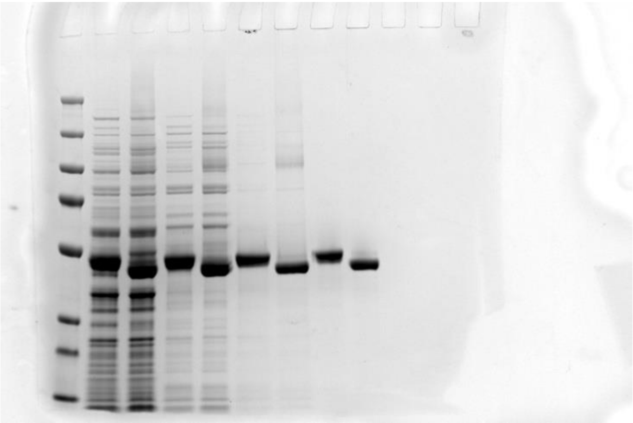

Blot 1:

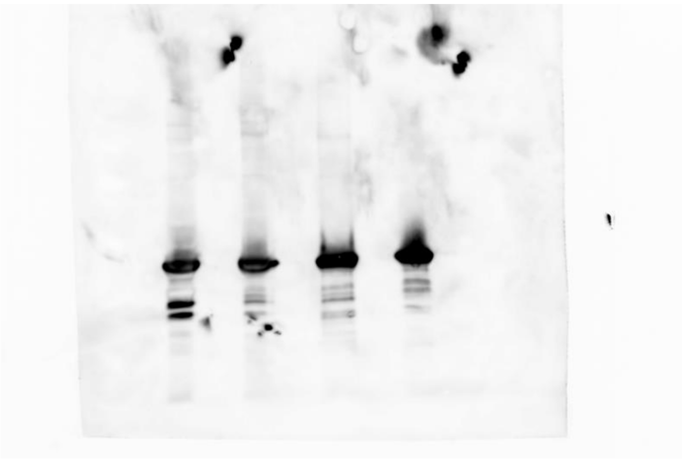

Blot 2:

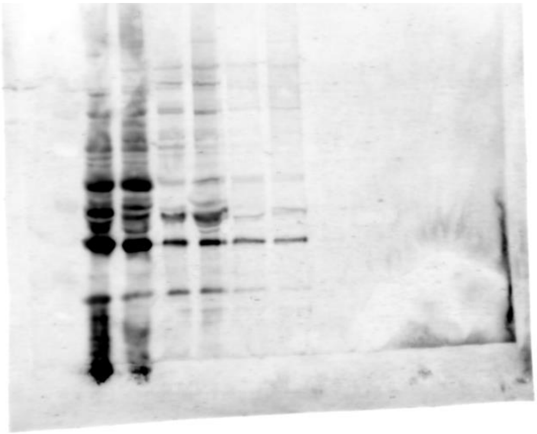

Supplementary Figure S7. Raw original image files for Figure 3b (SDS-PAGE and Western blot)

Supplementary Tables:

Supplementary Table S1. Column parameters for the purification of ProC6C

IEC Q HP Capturing	1 L	5 L
Resin	HiPrep Q HP (GE Healthcare)	HiPrep Q HP (GE Healthcare)
Dimensions	Prepacked 16/10	XK-26
Flow rate	5 ml/min	5 ml/min
Column Volume	20ml	79.6ml
Column Pressure	0.5 Mpa	0.6 Mpa
Buffer	Steps	Steps
A: 20mM HEPES, 5%Glucose, 50mM Sod. Borate, 10mM L-arginine, 1mM EDTA, pH6.5	Load: 0%<br>Wash: A+50mM NaCl<br>Wash: A+100mM NaCl<br>Elution: A+200mM NaCl<br>Strip: A+1M NaCl	Load: 0%<br>Wash: A+50mM NaCl<br>Wash: A+100mM NaCl<br>Elution: A+200mM NaCl<br>Strip: A+1M NaCl
Load volume	0.2L	1.5L
Load conductivity	8.23 mS/cm	8.58 mS/cm
Load pH	6.5	6.5
Eluate Volume	115 ml	320 ml
Eluate Conductivity	21.12 mS/cm	21.5 mS/cm
Eluate Peak Height	3000 mAU	3000 mAU
Eluate Peak Area	36674	98058

Capture Select HCP removal	1 L	5 L
Resin	Capture selectXL (GE Healthcare)	
Dimensions	Prepacked 5ml (5mlx3Columns)	XK-26
Flow rate	2.5ml/min	2.5ml/min
Column Volume	15ml	79.6ml
Column Pressure	0.3 Mpa	0.6 Mpa
Buffer	Steps	Steps
B: 20mM HEPES, 5% Glucose, 5mM L-arginine, 1mM EDTA, pH7.0	Load: 0%<br>Wash: B<br>Wash: B+140mM $MgCl_2$<br>Elution: B+700mM $MgCl_2$<br>Strip: B+2M $MgCl_2$	Load: 0%<br>Wash: B<br>Wash: B+140mM $MgCl_2$<br>Elution: B+700mM $MgCl_2$<br>Strip: B+2M $MgCl_2$
Load volume	920 ml	2560 ml
Load conductivity	5.02mS/cm	5.12 mS/cm
Load pH	7.0	7.0
Eluate Volume	125 ml	360 ml
Eluate Conductivity	75.95 mS/cm	75.25 mS/cm
Eluate Peak Height	890 mAU	1530 mAU
Eluate Peak Area	8034.2	14559

IEC Q HP Polishing	1 L	5 L
Resin	HiPrep Q HP (GE Healthcare)	HiPrep Q HP (GE Healthcare)
Dimensions	Prepacked 16/10	XK-26
Flow rate	4ml/min	4ml/min
Column Volume	20ml	79.6ml
Column Pressure	0.5 Mpa	0.6 Mpa
Buffer	Steps	Steps

C: 20mM HEPES, 5% Glucose, 1mM EDTA, pH8.0	Load: 0%<br>Wash: C+150mM NaCl Wash: C+270mM NaCl<br>Elution: C+310mM NaCl<br>Strip: C+1M NaCl	Load: 0%<br>Wash: C+150mM NaCl<br>Wash: C+270mM NaCl<br>Elution: C+310mM NaCl<br>Strip: C+1M NaCl
Load volume	1000 ml	2880 ml
Load conductivity	9.18 mS/cm	9.20 mS/cm
Load pH	8.0	8.0
Elutae Volume	50ml	190 ml
Eluate Conductivity	71.7 mS/cm	71.23 mS/cm
Eluate Peak Height	190 mAU	570 mAU
Eluate Peak Area	1555	1870
