# Supplementary material for: Preclinical development of a Pfs230-Pfs48/45 chimeric malaria transmission-blocking vaccine
Source: NPJ Vaccines. 2021 Oct 12;6:120. doi: 10.1038/s41541-021-00383-8 (PMC8511065; doi:10.1038/s41541-021-00383-8)
Supplement: Supplementary file 2 — Reporting Summary [file 41541_2021_383_MOESM2_ESM.pdf]

nature portfolio

Corresponding author(s): Michael Theisen, mth@ssi.dk

Last updated by author(s): Jul 19, 2019

Reporting Summary

Nature Portfolio wishes to improve the reproducibility of the work that we publish. This form provides structure for consistency and transparency in reporting. For further information on Nature Portfolio policies, see our Editorial Policies and the Editorial Policy Checklist.

Statistics

For all statistical analyses, confirm that the following items are present in the figure legend, table legend, main text, or Methods section.

n/a	Confirmed	
☐	☒	The exact sample size (n) for each experimental group/condition, given as a discrete number and unit of measurement
☐	☒	A statement on whether measurements were taken from distinct samples or whether the same sample was measured repeatedly
☒	☐	The statistical test(s) used AND whether they are one- or two-sided<br>*Only common tests should be described solely by name; describe more complex techniques in the Methods section.*
☒	☐	A description of all covariates tested
☒	☐	A description of any assumptions or corrections, such as tests of normality and adjustment for multiple comparisons
☐	☒	A full description of the statistical parameters including central tendency (e.g. means) or other basic estimates (e.g. regression coefficient) AND variation (e.g. standard deviation) or associated estimates of uncertainty (e.g. confidence intervals)
☒	☐	For null hypothesis testing, the test statistic (e.g. F, t, r) with confidence intervals, effect sizes, degrees of freedom and P value noted<br>*Give P values as exact values whenever suitable.*
☒	☐	For Bayesian analysis, information on the choice of priors and Markov chain Monte Carlo settings
☒	☐	For hierarchical and complex designs, identification of the appropriate level for tests and full reporting of outcomes
☒	☐	Estimates of effect sizes (e.g. Cohen's d, Pearson's r), indicating how they were calculated

Our web collection on statistics for biologists contains articles on many of the points above.

Software and code

Policy information about availability of computer code

Data collection	Quant Assay for Windows, Version V0.7.1.4
Data analysis	Prism version 9

For manuscripts utilizing custom algorithms or software that are central to the research but not yet described in published literature, software must be made available to editors and reviewers. We strongly encourage code deposition in a community repository (e.g. GitHub). See the Nature Portfolio guidelines for submitting code & software for further information.

Data

Policy information about availability of data

All manuscripts must include a data availability statement. This statement should provide the following information, where applicable:

- Accession codes, unique identifiers, or web links for publicly available datasets
- A description of any restrictions on data availability
- For clinical datasets or third party data, please ensure that the statement adheres to our policy

The datasets generated during and/or analysed during the current study are available from the corresponding author on reasonable request.

Field-specific reporting

Please select the one below that is the best fit for your research. If you are not sure, read the appropriate sections before making your selection.

☒ Life sciences ☐ Behavioural & social sciences ☐ Ecological, evolutionary & environmental sciences

For a reference copy of the document with all sections, see nature.com/documents/nr-reporting-summary-flat.pdf

Life sciences study design

All studies must disclose on these points even when the disclosure is negative.

Sample size	1st Mouse immunogenicity studies included n=6 for each group. 2nd Mouse immunogenicity study included n=5
Data exclusions	None
Replication	ELISA analysis of individual mouse sera were analzed in duplicate on the same plate in a single analysis.<br>SMFA was conducted from two separate study replicates on a single sera pool for each group in each analysis.<br>Hepatoycyte invasion assay was conducted in two separate study replicates in triplicate sample for each analysis.
Randomization	N/A
Blinding	None

Reporting for specific materials, systems and methods

We require information from authors about some types of materials, experimental systems and methods used in many studies. Here, indicate whether each material, system or method listed is relevant to your study. If you are not sure if a list item applies to your research, read the appropriate section before selecting a response.

Materials & experimental systems

n/a	Involved in the study	
☐	☒	Antibodies
☐	☒	Eukaryotic cell lines
☒	☐	Palaeontology and archaeology
☐	☒	Animals and other organisms
☒	☐	Human research participants
☒	☐	Clinical data
☒	☐	Dual use research of concern

Methods

n/a	Involved in the study	
☒	☐	ChIP-seq
☒	☐	Flow cytometry
☒	☐	MRI-based neuroimaging

Antibodies

Antibodies used	TBV antibody mAb 45.1 which is reported in public literature (provided by RUMC) and reacts to Epitope I of Pfs48/45. mAb45.1 is used to test proper folding of Pfs48/4t recombinant proteins via ELISA and WB. 2A10 AIV antibody which is reported in public literature (provided by RUMC) was used in hepatocyate invasion as positive control.
Validation	Antibodies are routinely generated and tested for their application as positive controls in ELISA and Hepatocyte invasion assay. All controls behaved as expected in our analysis. Each antibody has been used in previous studies and reported in peer reviewed literature. Citations may be found in the methods and reference section of the manuscript.

Eukaryotic cell lines

Policy information about cell lines

Cell line source(s)	human hepatoma cells (HC-04), ATCC MRA-975, reported in reference
Authentication	Cell lines were not authenticated, see references
Mycoplasma contamination	Cells were tested for mycoplasma and were negative.
Commonly misidentified lines (See ICLAC register)	None

Animals and other organisms

Policy information about studies involving animals; ARRIVE guidelines recommended for reporting animal research

Laboratory animals	female CD-1 mice (Janvier Labs, Denmark)
Wild animals	N/A
Field-collected samples	N/A
Ethics oversight	All animal procedures complied with national regulations and were approved by the ethics committee of the University of Copenhagen (approval number P 19-308) and the Radboud University Medical Center (approval number 2016-0020).

Note that full information on the approval of the study protocol must also be provided in the manuscript.
